# Non-canonical roles of *CFH* in retinal pigment epithelial cells revealed by dysfunctional rare *CFH* variants

**DOI:** 10.1016/j.stemcr.2024.11.015

**Published:** 2025-01-02

**Authors:** Sofie C.A. Brink, Louet Koolen, Caroline C.W. Klaver, Remko A. Bakker, Anneke I. den Hollander, Seba Almedawar

**Affiliations:** 1 Department of Ophthalmology, Radboud University Medical Center, Nijmegen, the Netherlands; 2 Department of Cardio Metabolic Diseases Research, Boehringer Ingelheim Pharma GmbH & Co. KG, Biberach, Germany; 3 Department of Epidemiology, Erasmus University Medical Center, Rotterdam, the Netherlands; 4 Department of Ophthalmology, Erasmus University Medical Center, Rotterdam, the Netherlands; 5 Institute of Molecular and Clinical Ophthalmology, University of Basel, Basel, Switzerland

**Keywords:** RPE, AMD, CFH, *in vitro* disease modeling, hiPSC

## Abstract

Complement factor H (*CFH*) common genetic variants have been associated with age-related macular degeneration (AMD). While most previous *in vitro* RPE studies focused on the common p.His402Tyr *CFH* variant, we characterized rare *CFH* variants that are highly penetrant for AMD using induced pluripotent stem-cell-derived retinal pigment epithelium (iPSC-RPE). Our results show that lower factor H (FH) levels are detected in AMD RPE, which potentially disrupt canonical and non-canonical roles of FH. Specifically, AMD RPE displays higher inflammation rate and a reduced set of differentially expressed genes compared to control RPE upon N-retinylidene-N-retinylethanolamine (A2E) and blue light challenge. Additionally, cholesterol efflux and photoreceptor outer segment (POS) phagocytosis defects, dysregulated complement levels, larger sub-RPE deposits, and increased mitochondrial stress were observed in AMD RPE. Thus, our study reveals new non-canonical roles for FH in regulating important RPE functions.

## Highlights

• Characterization of iPSC-RPE AMD model with rare highly penetrant *CFH* variants
• Cholesterol efflux defects reveal a new non-canonical role for *CFH* in RPE
• *CFH* variant iPSC-RPE displayed reduced transcriptional response to A2E and blue light
• *CFH* variant iPSC-RPE displayed POS phagocytosis defects and sub-RPE deposits

## Graphical abstract

**Figure:**
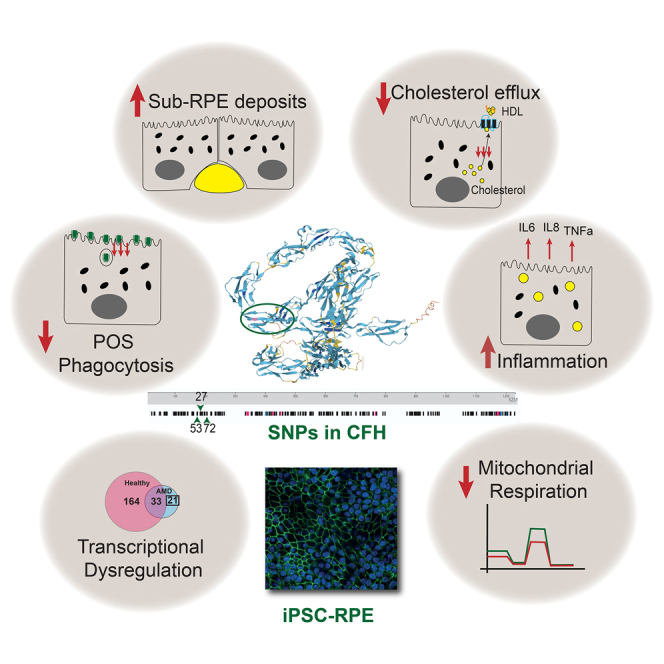

## Teaser

Almedawar and colleagues generated and characterized an iPSC-RPE model from patients with AMD with highly penetrant *CFH* variants. The model displays cholesterol efflux defects, as well as a reduced transcriptional response to A2E and blue light. They also observed POS phagocytosis defects, mitochondrial stress, and sub-RPE deposits similar to *CFH* H402Y risk models.

## Introduction

Age-related macular degeneration (AMD) is a degenerative disorder of the retina and retinal pigment epithelium (RPE)-choroid and is one of the leading causes of non-preventable vision loss in the elderly (Steinmetz et al., 2021). AMD is caused by a combination of environmental and genetic factors. Genome-wide association studies have identified many variants, in or near genes encoding components of the complement system, associated with altered AMD risk (Fritsche et al., 2016; Han et al., 2020). However, the effect of these genetic variants and the associated disease mechanisms are not fully understood. The most well-characterized and common variant is FH His402Tyr. However, other highly penetrant complement factor H (*CFH*) variants, causing early-onset AMD, have not yet been functionally characterized.

Factor H (FH) is a regulator of the complement system, which as part of its canonical role accelerates dissociation of C3b and Bb and acts as a cofactor for FI in cleaving C3b. Both functions inhibit the positive feedback loop of the alternative complement pathway (Pangburn et al., 1977; Weiler et al., 1976; Whaley and Ruddy, 1976). By binding to sialic acid and peptidoaminoglycans (GAG), FH is selectively increased on cell surfaces, concentrating its regulatory functions on C3b deposited on cells (Fearon, 1978; Kazatchkine et al., 1979; Meri and Pangburn, 1990; Pangburn and Müller-Eberhard, 1978). Ocular FH and the shorter splice variant factor H-like 1 (FHL1) are primarily derived from RPE-choroid and bipolar cells (Clark et al., 2014; de Jong et al., 2021; Khandhadia et al., 2013; Lukowski et al., 2019). In addition to its complement regulatory role, FH has non-canonical roles in RPE such as the regulation of nuclear factor κB and mTOR pathways, protection from lipid oxidation products, protection from mitochondrial dysfunction and oxidative stress, photoreceptor outer segment (POS) phagocytosis, and possibly within the ubiquitin proteasome system (Armento et al., 2023; Schäfer et al., 2021).

To understand the role of genetic variants in disease mechanisms and test the effect of new treatments, accurate disease models and relevant cellular phenotypes are needed. AMD patient induced pluripotent stem cells (iPSCs) can be derived from somatic cells, and differentiation can then be directed to RPE (Regent et al., 2019). iPSC-derived RPE (iPSC-RPE) cells are phenotypically similar to native RPE (Kokkinaki et al., 2011) and can reproduce disease phenotypes *in vitro* (Cai et al., 2021; Chang et al., 2014; Golestaneh et al., 2016; Gong et al., 2020; Hallam et al., 2017; Keir et al., 2017; Saini et al., 2017; Yang et al., 2014).

Here, we aimed to create and evaluate iPSC-RPE models of patients with AMD with highly penetrant *CFH* variants: *CFH* c.607-610dupCCAA (p.Lys204Thrfs^∗^26), *CFH* c.550del (p.Ile184Leufs^∗^33), *CFH* c.524G>A (p.Arg175Gln), hereafter referred to as Lys204fsX, Ile184fsX, and Arg175Gln. To characterize the differentiated RPE cells, we subjected them to a range of assays to determine the quality of the RPE and to identify the disease-related phenotypes, thereby revealing known and new non-canonical roles for *CFH* in the RPE.

## Results

### *CFH* expression is reduced in patient iPSC-derived RPE cells harboring *CFH* SNPs

**Figure 1:**
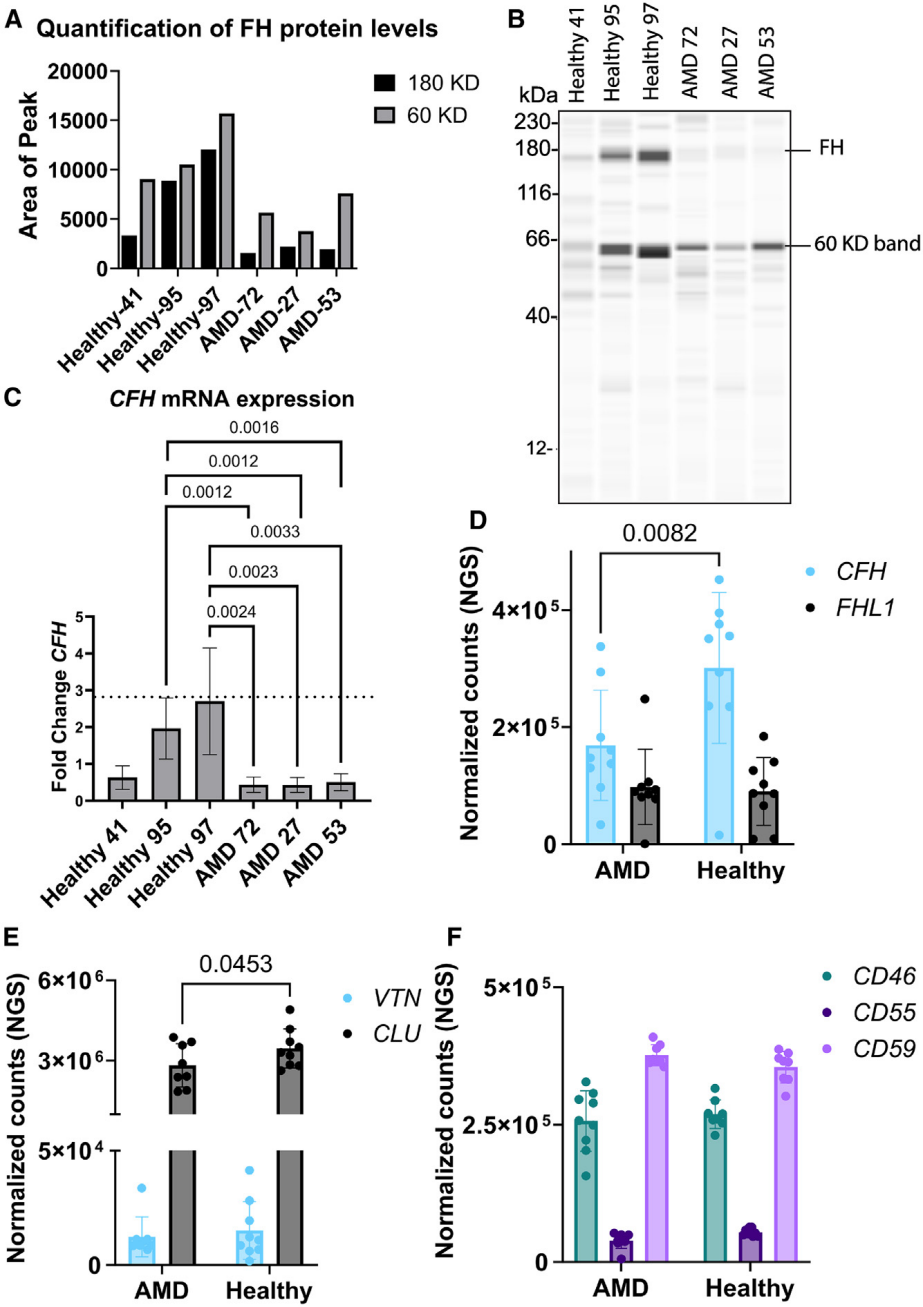
CFH expression levels are reduced in AMD RPE carrying rare CFH variants (A) Quantification of western blot in (B). (B) Western blot analysis of FH protein expression. *n* = 3 pooled wells, no technical repeats. (C) Quantitative PCR analysis of CFH mRNA expression in healthy vs. AMD RPE cells. *N* = 3 differentiation rounds and 3 pooled wells per line. Error bars represent mean ± SD. Ct values were subtracted from GAPDH housekeeping gene (ΔCt) and then from mean ΔΔCt of healthy RPE. (D) CFH and FHL1 mRNA expression from NGS dataset. *N* = 3 differentiation rounds. Error bars represent mean ± SD. (E and F) mRNA expression levels from the NGS dataset of CRPs. *N* = 3 differentiation rounds. Error bars represent mean ± SD.

RPE cells were differentiated from iPSC lines that have been previously described: iPS17-00041, iPS17-00095, iPS17-00097, iPS18-00072, iPS19-00027, and iPS19-00053, hereafter referred to as lines 41, 95, 97, 72, 27, and 53, respectively (Koolen et al., 2022a, 2022b). Clinical ocular manifestations, genetic risks, and mutations of healthy and *CFH* rare variant carrier AMD donors are summarized in Figure S1 and Table S1. The family pedigree reflecting genotype and disease history is shown in Figure S2. Prior to functional assays, the quality of differentiated RPE cells was evaluated. Immunofluorescence staining of RPE cells shows polarized expression of bestrophin in the basolateral side, MERTK and Ezrin in the apical side, tight junctions’ marker ZO1 in the apical-lateral side, and MITF in the nuclei (Figure S3). RNA sequencing (RNA-seq) analysis and qPCR show comparable expression of these genes (Figure S4). Interestingly, we observed on the RNA and protein levels lower *CFH*/FH in all AMD RPE and in healthy RPE line 41 (Figures 1A–1C). Another 60 kDa band was detected and seemed to be reduced in AMD lines (Figure 1B). Next-generation sequencing (NGS) analysis showed a reduction in *CFH* but not *FHL1* mRNA expression levels (Figure 1D). In addition to CFH, we checked mRNA levels of other complement regulatory proteins (CRPs) in our NGS dataset. We observed only a significant difference in *CLU* mRNA expression levels but not in *VTN*, *CD46*, *CD55*, and *CD59* between healthy and AMD RPE (Figures 1E and 1F).

### AMD RPE shows differential response to A2E and blue light challenge

**Figure 2:**
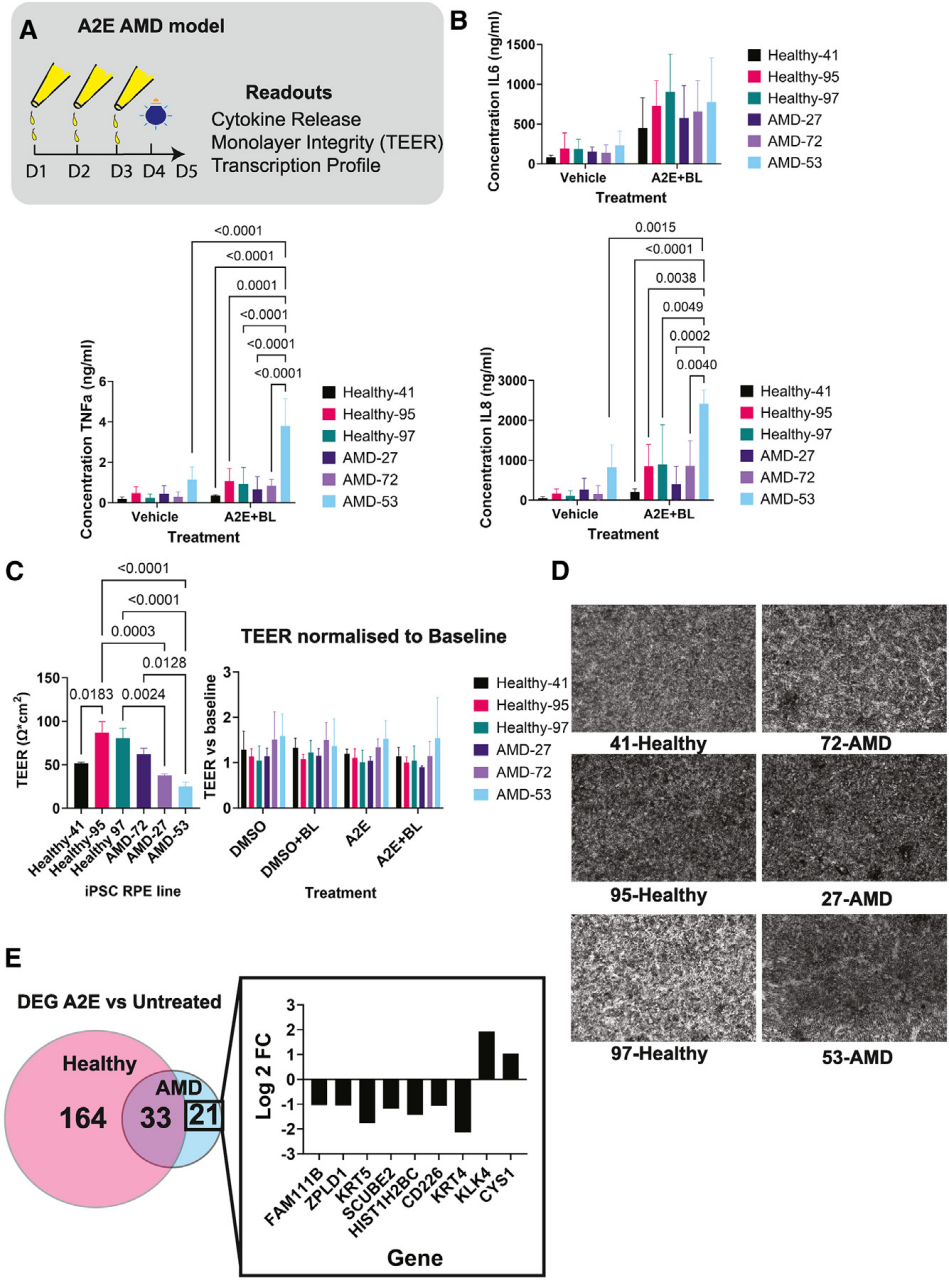
A2E aging model of RPE cells (A) The experimental plan and readouts. (B) MSD ELISA measurements of cytokines released from aged RPE cells. *N* = 3 differentiation rounds and 3 wells per line and condition. Error bars represent mean ± SD. (C) Baseline TEER measurement and TEER changes following A2E and light damage. *N* = 3 differentiation rounds and 3 wells per line and condition. Error bars represent mean ± SD. (D) Representative bright-field images of RPE cells from different donors. (E) The number of significant (*p* value <0.01) differentially expressed genes (DEGs) in A2E and light-treated vs. untreated conditions in AMD and control RPE. The graph represents the uniquely protein-coding DEG in AMD RPE. *N* = 3 differentiation rounds. Information for the other DEGs can be found in Figure S5.

As described by Yang et al. (2014), iPSC-RPE cells were aged using N-retinylidene-N-retinylethanolamine (A2E) and blue light challenge (Figure 2A). All lines displayed a similar increase in secreted IL-6 levels upon treatment with A2E and blue light. Interestingly, AMD RPE line 53 showed increased IL-8 and TNF-α secretion without challenge and a more significant increase upon A2E and blue light challenge (Figure 2B). AMD RPE had lower basal transepithelial electrical resistance (TEER) compared to control. However, A2E did not seem to affect the TEER or the morphology of AMD and control RPE (Figures 2C and 2D). Interestingly, RNA-seq displayed differences in the response to A2E between AMD and control RPE. While only 21 genes were specifically differentially expressed in AMD lines, 164 genes were specifically differentially expressed in control RPE and only 33 genes were commonly differentially expressed in both AMD and control RPE (Figures 2E and S5).

### Metabolic changes in AMD RPE observed in the Seahorse assay

**Figure 3:**
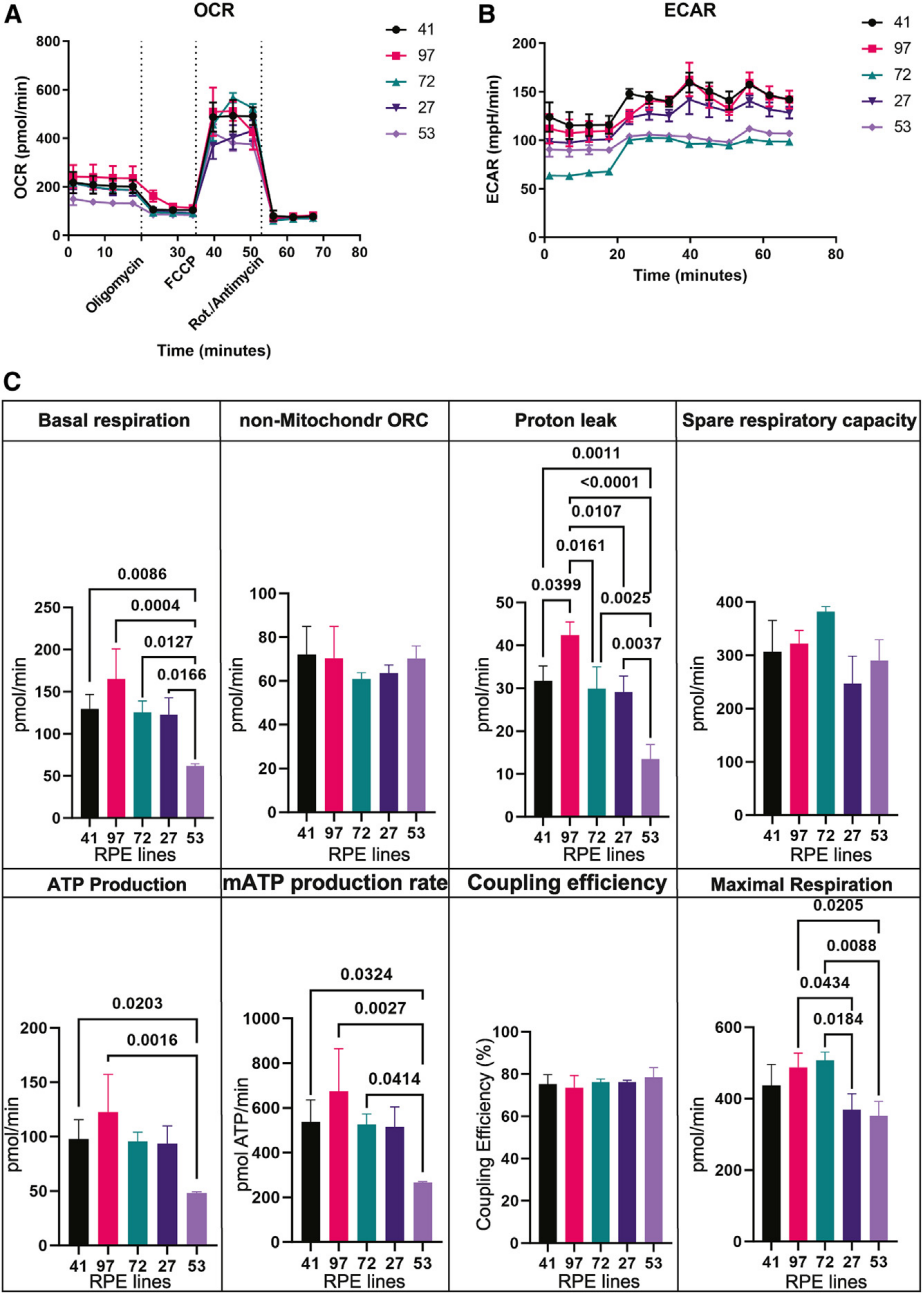
Seahorse assay showing metabolic changes in AMD RPE. (A) OCR over the entire course. Error bars represent mean ± SD. (B) ECAR over the entire course. Error bars represent mean ± SD. (C) Calculated values derived from the measurements in (A) and (B). Refer to the methods sections for the calculation methods. *N* = 3 differentiation rounds and 3 wells per line and condition. Error bars represent mean ± SD.

Metabolic defects have been previously described in the common risk variant (Sharma et al., 2021). To determine the effect of the rare *CFH* variants on metabolism, a mitochondria stress test using the Seahorse system was performed. Upon initial evaluation, iPSC-RPE lines 53 and 27 displayed clear defects in both extracellular acidification rate (ECAR) and oxygen consumption rate (OCR) (Figures 3A and 3B). Detailed examination revealed that AMD lines did not appear to have a coupling deficiency. Additionally, minor defects were observed in non-mitochondrial OCR in lines 72 and 27. Similarly, lines 27 and 53 showed minor defects in spare respiratory capacity. However, line 53 displayed a lower maximal respiration rate, basal respiration rate, ATP production rate, and decreased proton leak (Figure 3C), while line 27 showed only a lower maximal respiration rate (Figure 3C).

### AMD RPE shows defects in cholesterol efflux

**Figure 4:**
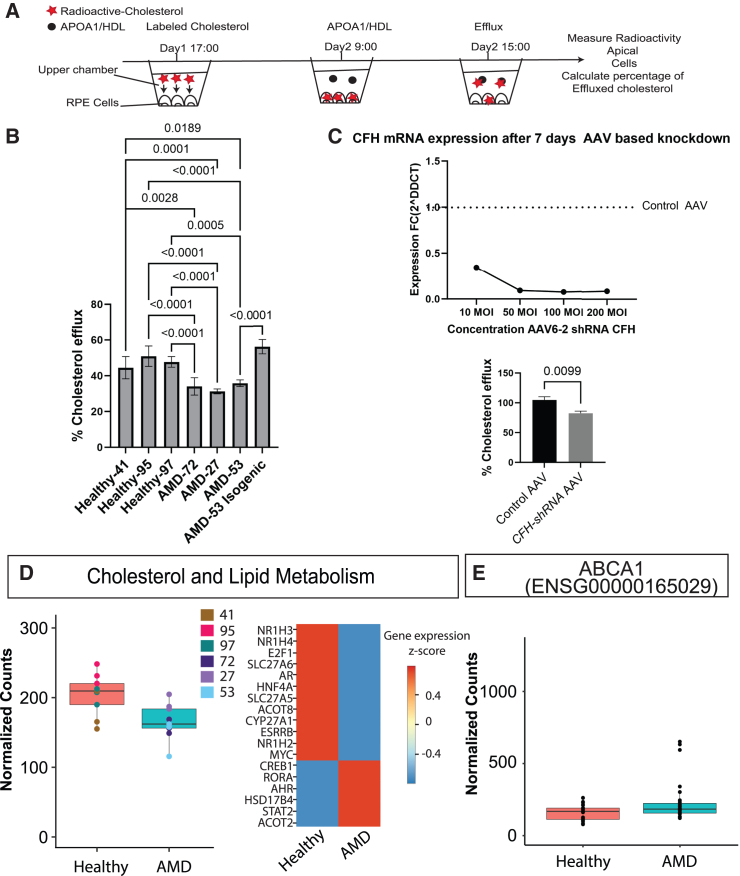
AMD RPE shows defects in lipid metabolism and cholesterol efflux (A) Experimental setup: radioactively labeled cholesterol was added on day 1, after 16 h HDL was added, at 22 h radioactivity was measured in supernatant and cells. (B) % cholesterol efflux in iPSC-RPE. *N* = 3 differentiation rounds and 3 wells per line and condition. Error bars represent mean ± SD. (C) High CFH knockdown (KD) efficacy was shown already at 50 MOI 7 days post transfection. CFH KD reduced cholesterol efflux. *N* = 3 wells per condition. (D) RNA-seq displayed a reduced expression of cholesterol and lipid metabolism-related genes, listed in the heatmap, in AMD lines vs. controls. *N* = 3 differentiation rounds. Error bars represent mean ± SD. (E) ABCA1 expression was unaffected in AMD lines. *N* = 3 differentiation rounds. Error bars represent mean ± SD.

Previous literature evidence showed a role for FH in stimulating cholesterol efflux in THP1 cells (Nissilä et al., 2018). Thus, we investigated the effect of the different *CFH* variants on cholesterol efflux in RPE cells (Figure 4A). A marked decrease in cholesterol efflux was seen in AMD RPE when compared to control RPE. The cholesterol efflux defect could be rescued in AMD line 53 by correcting the *CFH* mutation (Figure 4B). Line 27 was also homozygous for the ABCA1 risk allele (AA) for SNP rs2740488, whereas lines 72 and 53 were heterozygous. Despite the *ABCA1*-risk SNPs, *ABCA1* expression was unaffected in AMD RPE (Figure 4E). Knockdown of *CFH* using AAV expressing *CFH shRNA* reduced cholesterol efflux in primary human RPE cells similar to AMD RPE cells (Figure 4C). In addition, all AMD lines had reduced expression of genes involved in lipid and cholesterol metabolism (Figure 4D).

### Sub-RPE deposit formation after prolonged culture of AMD RPE cells

**Figure 5:**
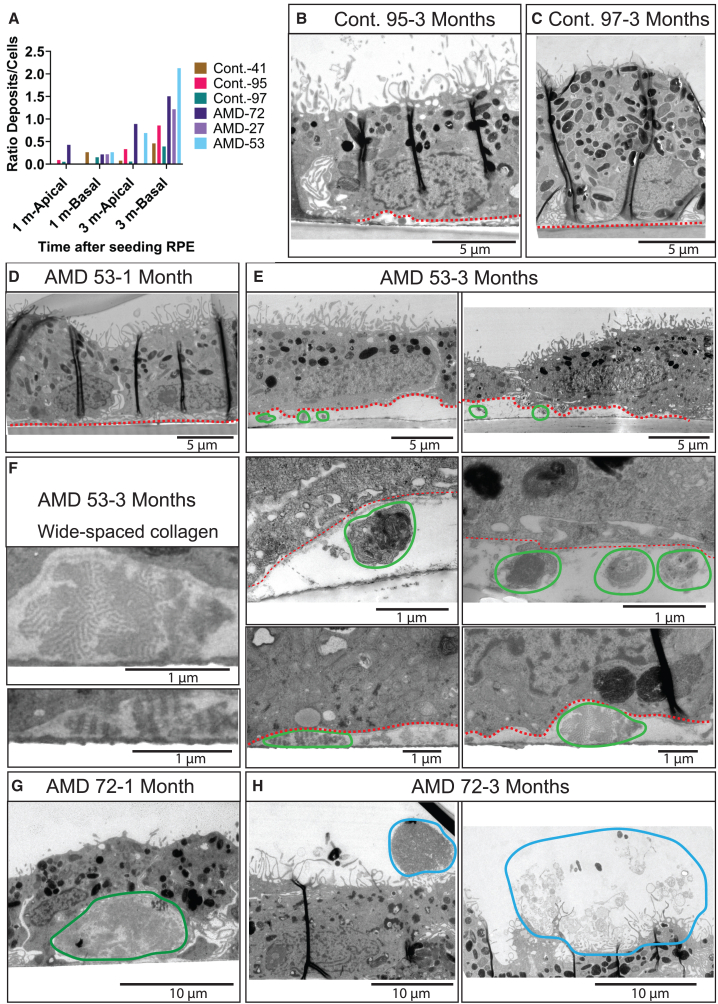
Ultrastructural analysis of iPSC-RPE reveals AMD RPE deposit formation after prolonged culture Deposits on the apical side are marked with blue line, while basal side deposits are marked with a green line. Red dotted line shows the separation between RPE and the supporting membrane. (A) Quantification of the deposits 1 or 3 months after seeding RPE cells on transwells after passage 2 (P2). (B) A representative picture of control RPE 95 after 3 months of seeding. Note the small basal deposits found in some of the fields. (C) Control RPE 97 with no basal deposits. (D) AMD RPE 53 after 1 month of seeding with no basal deposits. (E) Sample pictures of deposits found in AMD RPE 53 after 3 months. (F) Magnification of the collagen deposits in (E). (G) Big basal deposits observed in AMD RPE 72. (H) Big apical deposits observed in AMD RPE 72.

Drusen formation is an important clinical hallmark of AMD. As expected, drusen was observed in our AMD donor eyes (Figure S1). The observed defects in cholesterol efflux and increase in inflammation and mitochondrial stress in AMD RPE suggested that lipid metabolism might be slowed down leading to deposit formation. Thus, we examined the ultrastructure in AMD RPE after prolonged culture using electron microscopy (Figure 5). We observed polarized RPE cell morphology with apical pigments and microvilli, and basal nuclei and membranous foldings in control RPE and in some of the AMD RPE at 1 month of culture. AMD line 53 displayed circular or collagen deposits with thickening of the electron sparse area between the insert membrane and RPE layer after 3 months compared to 1 month of culture. Line 72 already displayed an elliptical deposit on the basolateral side after 1 month, with additional apical deposits. Control lines displayed fewer and smaller deposits after 3 months of culture.

### AMD RPE shows defects in POS phagocytosis

**Figure 6:**
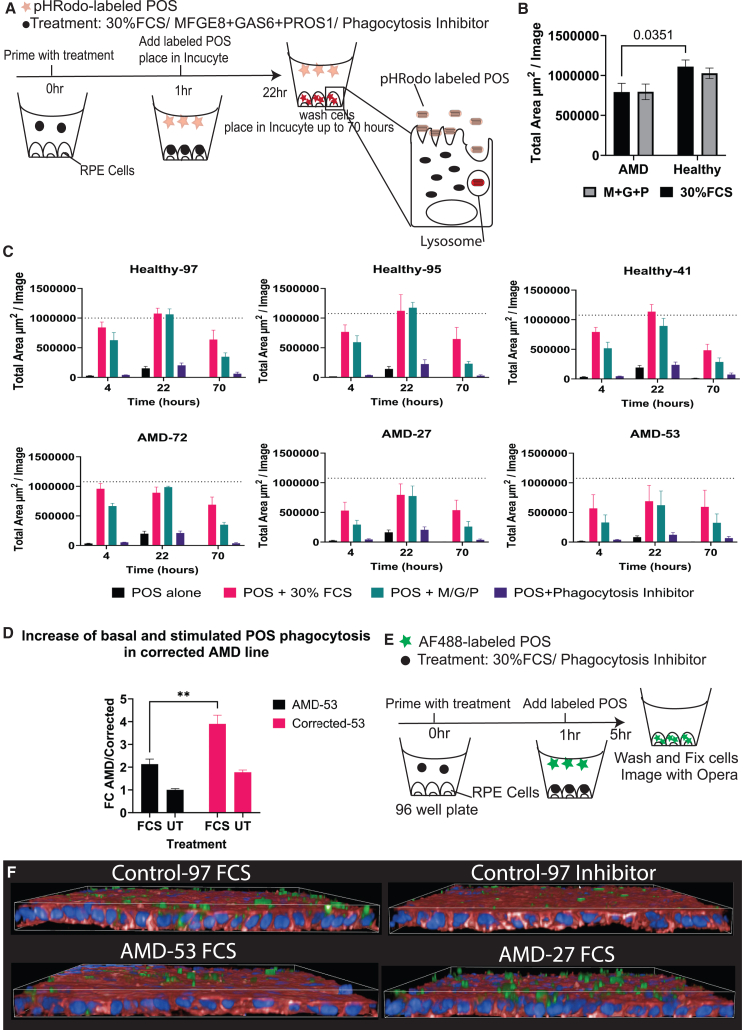
AMD RPE shows defects in POS phagocytosis (A) The experimental setup of live imaging of RPE cells challenged with pHRodo using Incucyte. pHRodo-labeled POSs are weakly fluorescent at high pH and gain higher fluorescence as they become internalized and exposed to lower pH. (B) Average of POS phagocytosis in AMD lines vs. healthy with FCS or MFGE8+GAS6+PROS1. *N* = 3 differentiation rounds and 3 wells per line and condition. Error bars represent mean ± SD. (C) Analysis of live imaging of internalized POS at different timepoints after pHRodo-labeled POS addition. AMD RPEs show defects in POS internalization. *N* = 3 differentiation rounds. Error bars represent mean ± SD. (D) Correction of the CFH mutation in line 53 led to 2-fold increase of POS phagocytosis at 22 h. *N* = 3 differentiation rounds and 3 wells per line and condition. Error bars represent mean ± SD. (E) The experimental setup of confocal imaging of fixed RPE cells challenged with AF488. (F) Confocal images of RPE cells labeled with Phalloidin (red) and Hoechst (blue) after fixation. Bound and internalized POSs (green) were observed upon FCS treatment in control RPE and no internalized POSs were observed in control RPE treated with the phagocytosis inhibitor. POS remained bound to the apical surface in the presence of the inhibitor. Very few internalized POSs were observed in lines 27 and 53 in the presence of FCS, instead, POSs were mostly bound to the apical surface. *N* = 3 wells per line and condition.

POS phagocytosis is a major function of RPE, which has been previously shown to be reduced in RPE cells homozygous for the *CFH* His402Tyr risk allele (Sharma et al., 2021). To determine whether our AMD RPE would have a similar defect, we performed a live imaging-based POS phagocytosis assay (Figure 6A). All tested AMD RPE lines showed decreased POS internalization. However, lines 27 and 53 showed the most severe defect in POS internalization (Figure 6C). Correction of the *CFH* mutation in line 53 led to a 2-fold increase of POS phagocytosis at 22 h. POS degradation after 70 h did not seem to be affected in AMD RPE in the presence of the phagocytosis ligands MFGE8, GAS6, and PROS1. However, POS degradation defect was observed in AMD RPE in the presence of FCS (fetal calf serum). To confirm the phagocytosis defect in lines 27 and 53, we performed confocal imaging on fixed RPE following challenge with AF488-labeled POS for 5 h (Figure 6E). We observed bound and internalized POS in control RPE, upon FCS treatment, and almost no internalized POS in the presence of the phagocytosis inhibitor, or absence of phagocytosis stimulators (FCS or MFGE8+GAS6+PROS1). In contrast, very few internalized POSs were observed in AMD RPE 27 and 53 despite the presence of phagocytosis stimulators (Figure 6F).

### *CFH* variant leads to larger MAC deposits on the apical surface of RPE cells

**Figure 7:**
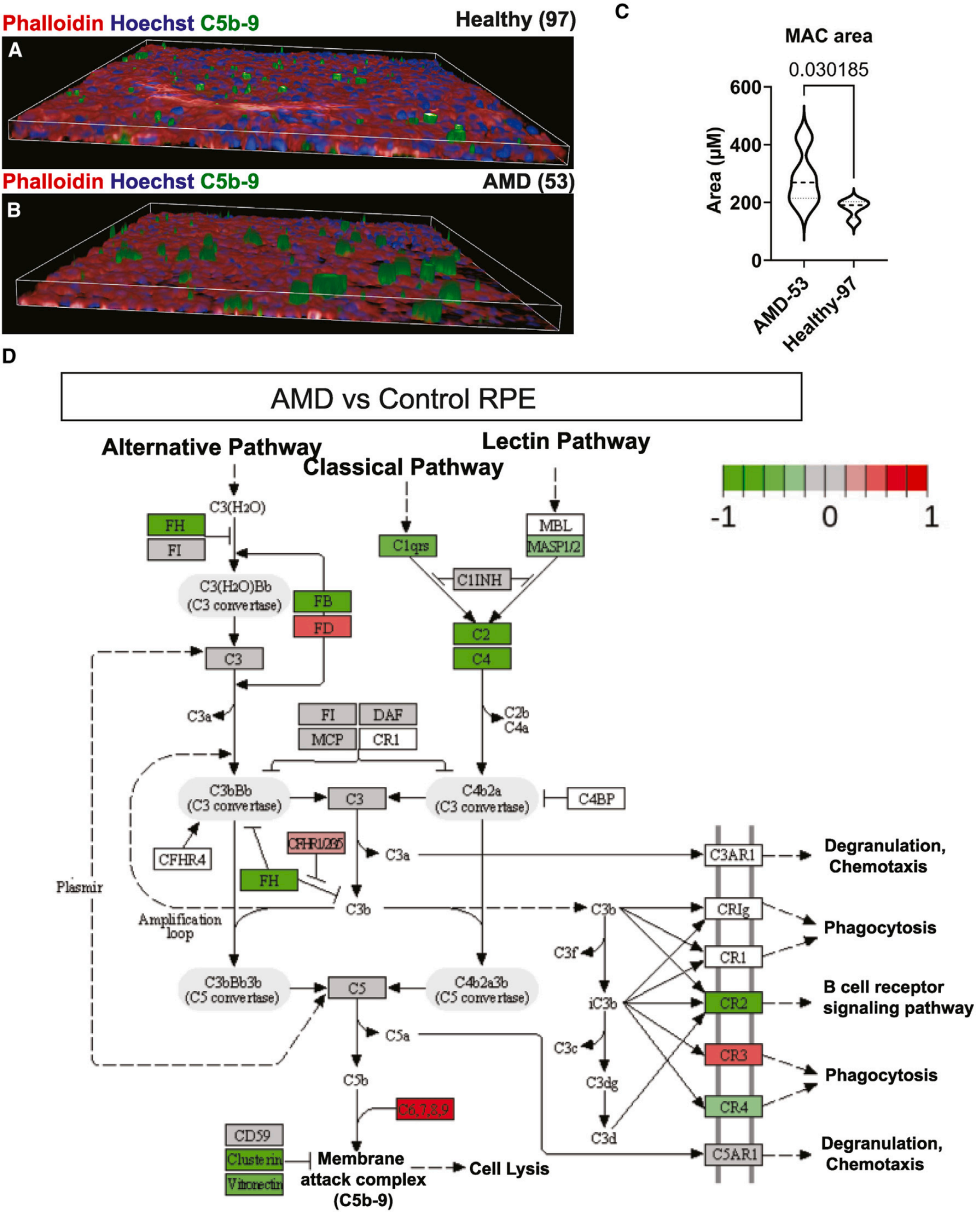
AMD RPE complement dysregulation (A and B) AMD RPE 53 displays larger MAC clusters than (B) control RPE 97. (C) Quantification of the images in (A) and (B). Due to the larger clusters, the total MAC area is increased. *N* = 3 wells per line. (D) RNA-seq changes in expression of the complement pathway in AMD versus control RPE. *N* = 3 differentiation rounds. RNA-seq changes in expression of the complement pathway in AMD RPE and control RPE under A2E challenge vs. untreated can be found in Figure S6. ^∗^ *p* value = 0.03.

Since FH modulates complement, we reasoned that lower FH levels must have an effect on complement factor expression or secretion and on MAC (membrane attack complex) deposition. To test this, we measured C3 and C5 secretion in AMD and control RPE using ELISA (Figure S4C). Interestingly, we observed that C5 is barely secreted in the RPE supernatant. However, secreted C5 was slightly elevated in AMD RPE. In contrast, C3 was abundant in the supernatant, but less C3 was detected in AMD RPE compared to control. In the absence of endogenous C5 secretion, we expected no detectable MAC deposition without external C5. For this purpose, we treated RPE cells with serum and observed MAC deposition using immunofluorescence labeling and confocal imaging. While the number of MAC deposits on the RPE was similar in control and AMD RPE, the area of MAC deposits in AMD RPE 53 was larger than that in control RPE 97 (Figures 7A–7C). Finally, RNA-seq Kyoto Encyclopedia of Genes and Genomes (KEGG) pathway analysis showed decreased *CFH* expression in AMD RPE when compared to control regardless of the A2E and blue light challenge (Figure 7D). In unchallenged RPE, some components of the complement pathway were upregulated such as *CFHR1/2/3/5* and *C6*, *C7*, *C8*, and *C9* and others were downregulated including *CFB* and *CLU* (Figure 7D). However, in the presence of A2E and blue light, all complement components were downregulated in control and AMD RPE (Figure S6). Expression of CRPs, other than *CFH*, including *CD46*, *CD55*, and *CD59* was not significantly different in AMD RPE compared to control regardless of A2E challenge (Figures 1D and 1E).

## Discussion

In this work, we characterized and performed functional studies with iPSC-RPE cells harboring rare but highly penetrant *CFH* variants. RPE line 53, with an average GRS (genetic risk score) score of 0.533 and a heterozygous *CFH* missense mutation, consistently showed reduced *CFH* levels, increased sensitivity to A2E, reduced TEER, increased mitochondrial stress, reduced POS phagocytosis, reduced cholesterol efflux, sub-RPE deposit accumulation, and enlarged MAC on the apical surface of RPE. The mutation (p.Arg175Gln) is located within the C3b binding domain 3 (Rodriguez et al., 2014) and might interfere with the binding of *CFH* to C3b, which regulates signaling amplification upon complement activation. Alternatively, the mutation can be dominant negative or can interfere with protein folding and stability, which might signal the protein to degradation.

RPE line 27, derived from 71-year-old male with a high GRS score of 2.927 and a heterozygous frameshift mutation leading to a predicted truncated FH protein, showed defects in some of the assays. Specifically, we observed reduced FH levels, increased sensitivity to A2E, reduced TEER, increased mitochondrial stress, sub-RPE deposit accumulation, reduced POS phagocytosis, and reduced cholesterol efflux. Enlarged MAC on the apical surface of the RPE was not observed in the time frame of this study. The truncated protein presumably contains the first three domains of FH, which might still bind to C3b but might be unstable and signaled to degradation, which might explain the observed phenotype.

RPE line 72, derived from a 60-year-old female with a high GRS score of 1.491 and a heterozygous frameshift mutation leading to a predicted truncated FH protein, showed reduced FH levels, increased sensitivity to A2E, reduced TEER, lower cholesterol efflux, and increased deposit formation below and above RPE cells. However, less severe phagocytosis defect and mitochondrial stress were observed, and no enlarged MAC deposits were detected. Similar to line 27, the truncated protein presumably contains the first three domains of FH, which might still bind to C3b but might be unstable and signaled to degradation, which might explain the observed phenotype.

One of the healthy control lines (41) unexpectedly displayed a slight reduction in FH levels compared to the other two control lines. While this line came from a healthy donor, it was heterozygous for the His402Tyr SNP, which might have contributed to the observed lower FH expression despite previous reports, which show that His402Tyr does not affect *CFH* expression (Ebeling et al., 2021). Nevertheless, we decided to keep this line as control to distinguish the effect of the newly characterized variants from the common His402Tyr variant.

While examining FH levels via western blot, a smaller band with reduced levels in AMD RPE compared to control was detected. We suspect that this band might correspond to FHL1 since the antibody we used detects amino acids 130–180 in FH, which aligns with the FHL1 amino acid sequence. However, we could not observe the same reduction at the mRNA level, and FHL1 is only 43 kDa (Schwaeble et al., 1987). Additionally, we verified the sequences of other proteins homologous to FH including FHR1-5; however, their amino acid sequence did not align with that amino acid detected by the antibody. Thus, the nature of this additional band still needs to be verified.

Despite the heterogeneity in the observed phenotypes across patients with AMD, all AMD RPE lines showed reduced cholesterol efflux and downregulation of lipid metabolism pathways. This, together with the increased size of deposits observed in those cells, suggests a non-canonical role for FH in lipid metabolism in RPE. A similar role has been described for FH in THP-1 macrophages (Nissilä et al., 2018).

We could identify wide-spaced collagen as one of the most common deposits in AMD RPE, which correlates with the pathological role of these deposits described during AMD development (Albert et al., 2022). Interestingly, similar collagen deposits have been observed *in vitro* upon prolonged C3a treatment (Fernandez-Godino and Pierce, 2018), supporting a strong link between complement dysregulation and drusen formation.

Defects in POS phagocytosis in AMD RPE suggest a role for FH during POS phagocytosis. Comparable findings have been previously observed by Sharma et al. (2021) using RPE homozygous for the FH His402Tyr risk allele. Despite lower FH levels in line 41, no phagocytosis defects were observed. This suggests that factors other than expression contribute to lower phagocytosis, such as protein folding or interaction with other proteins.

AMD RPE had lower basal TEER compared to control. This is in line with previous observations (Sharma et al., 2021). Aging with A2E did not seem to affect TEER or the morphology of RPE. However, it led to increased cytokine secretion. Interestingly, AMD RPE line 53 displayed a stronger IL-8 and TNF-α release with and without A2E, which can induce mitochondrial damage (Schulze-Osthoff et al., 1992). This explains the lower basal respiration and ATP production detected in the mitochondrial stress test in those RPE cells. Similar findings have been previously reported using FH His402Tyr risk RPE cells (Ebeling et al., 2021).

When comparing unchallenged AMD and control RPE, the expression of some components of the complement pathway, such as *CFHR1/2/3/5*, was upregulated. The cells might upregulate *CFHR1/2/3/5* to compensate for the lower FH levels. Additionally, the increase in *C6*, *C7*, *C8*, and *C9* expression and the decrease in *CLU* and *VTN* expression might explain the larger MAC complexes observed in AMD RPE upon serum challenge. This is in line with previous reports, which show that *CFH* His402Tyr iPSC-RPE displays larger MAC staining clusters (Cerniauskas et al., 2020). C5b9 has been mostly observed in drusen below RPE cells in AMD postmortem eyes. This is probably due to the systemic source of C5, which we confirm in this study, as we show that RPE does not produce C5 locally. C5b9 within or above RPE cells has been observed in degenerating RPE (Anderson et al., 2002; Bonilha et al., 2020; Cerniauskas et al., 2020; Georgiannakis et al., 2015) probably due to compromised blood-retina barrier and leakage of complement components from the systemic circulation. In this paper, we treat RPE cells with external complement source (serum) from the apical side leading to apical formation of C5b9 containing MAC, which resembles the clinical pictures described in degenerating RPE *in vivo*. Although *C3* mRNA expression was unaffected in AMD RPE, C3 secretion in the supernatant was reduced in AMD RPE compared to control. Interestingly, C5 expression was barely detected in both control and AMD RPE. However, a slight increase in the secreted C5 was observed in AMD RPE. After A2E challenge, *C3* RNA expression along with other complement components was unexpectedly downregulated in both control and AMD RPE. It should be noted that we did not measure activation or degradation products, which would have provided more functional information. Previous findings showed that high-His402Tyr-risk RPE challenged with A2E-rich *Abca4*^−/−^ POS causes similarly lower FH and CRP levels (Radu et al., 2014). How A2E results in reduced complement factors expression is currently unclear.

Interestingly, AMD RPE had a significantly lower number of differentially expressed genes after A2E exposure compared to control suggesting a reduced adaptability to stress. The relevance of AMD RPE unique differentially expressed genes (*FAM111B ZPLD1*, *KRT5*, *KRT5*, *SCUBE2*, *CD226*, *KRT4*, *KL4*, and *CYS1*) to the eye or AMD is generally unknown, except for *HIST1H2BC*, which is downregulated in AMD RPE upon A2E challenge, and has previously been shown to be downregulated in aged mouse RPE (Dubey et al., 2024).

Our study has several limitations. First, while control donors were phenotyped at the time of donation, it is unknown if controls would develop AMD later in life. Second, clinical studies show the involvement of several tissues in AMD pathogenesis including choroid. Thus, more complex models that include choroid-like tissue should be used in future studies. Finally, the iPSC model limits throughput relative to clinical studies. As such, generalizing to AMD overall would require confirmation of *in vitro* results in population studies.

In summary, our results indicate various phenotypes in the studied rare *CFH* variant RPE cells; some are novel such as the cholesterol efflux defect, while others are in line with previously described phenotypes in His402Tyr risk RPE, including POS phagocytosis defects, mitochondrial stress, and deposit formation. The RNA-seq data revealed two unexpected findings. First, upon treatment with A2E and blue light, AMD RPE showed a reduced number of differentially expressed genes compared to control, suggesting an inability to respond to insult. Second, A2E and blue light treatment reduced complement expression levels in both control and AMD RPE. While most reports in advanced AMD have shown complement upregulation under oxidative stress, our results suggest complement might be downregulated in the early stages of AMD where A2E-rich lipofuscin is still detected in patients’ fundus images, which highlights the complexity of complement regulation in AMD.

## Experimental procedures

See the supplementary methods for additional methods.

### iPSC source and differentiation to RPE

#### iPSC source

The donor selection, reprogramming, and characterization of the iPSC lines were described previously (Koolen et al., 2022a, 2022b). The use of iPSC within this study was approved by the human biospecimen office at Boehringer Ingelheim. In summary, three individuals over 70 years old without AMD and three patients with AMD with a rare highly penetrant mutation in *CFH* were selected from the European Genetic Database (EUGENDA).

#### Isogenic control generation

Isogenic controls were created for AMD RPE line 53 using CRISPR-Cas9. CAS9/12 was incubated with the gRNAs for 20 min at room temperature to prepare the RNP complex. iPSCs were digested to single cells using TrypLE and following centrifugation resuspended in the P3 primary Cell 4D-nucleofector X kit (Lonza) nucleofection solution containing the HDR template and corresponding RNP complex and transferred to a nucleovette strip well. Program CA-137 was used for nucleofection. After a 10-min incubation at room temperature, the cells were transferred to a Matrigel-coated 24-well plate with E8 Flex and incubated overnight at 37°C, 5% CO_2_. Once confluent, 500,000 cells were seeded as single cells on 10 cm Matrigel-coated dishes. In total, 48 clones were picked and seeded on 24-well plates. All clones were then sequenced to verify the gene editing. Successfully edited clones were expanded for storage and characterized for undifferentiation markers, trilineage differentiation, and mycoplasma presence.

### RPE differentiation

RPE differentiation was based on Regent et al. (2019). iPSCs were plated on Matrigel (Corning, 354230) and cultured in E8 Flex (Gibco, A2858501). Upon 80% confluency medium was replaced by RPE differentiation medium containing high glucose DMEM (79% [v/v]), KnockOut SR (20% [v/v], Gibco, 10828-28), non-essential ammino acids (1% [v/v], Sigma, M7145) and primocin (0.1 mg/mL, InvivoGen, Ant-pm2). Time-point-specific additives were used to ensure differentiation toward RPE: the first 7 days nicotinamide (10 mM, Sigma, N0636-100G), the next 7 days activin A (100 ng/mL, Sigma-Aldrich, H4666), and finally from 14 days differentiation onward CHIR 99021 (3 μM, Tocris Bioscience, 4423). RPE was initially passaged after 42 days using TrypLE Select (Gibco, 12563-029). RPE was seeded on transwell culture inserts coated with Matrigel in FMN medium (DMEM/F-12 [Gibco, 11320-074], N2 supplement [1% (v/v), Gibco, 17502-048], 1% [v/v] non-essential amino acids, and 0.1 mg/mL primocin). After 3 weeks, the RPE was passaged again to the desired culture vessel for experiments.

### POS phagocytosis evaluation using Opera Phoenix and Incucyte

POS isolation and POS labeling can be found in supplementary methods. Cells were primed with either 30% FCS or 2.5 μg/mL MFGE8 (R&D, 2767-MF), GAS6 (R&D, 885-G5), and PROS1 (R&D, 9489-PS-100) for 1 h at 37°C 5% CO_2_. To inhibit POS phagocytosis, 1 μM Phagocytosis Inhibitor BMS-777607 (Sigma, BM0019) was added to the priming mix. Meanwhile, POSs were labeled with AF488 for Opera high-throughput microscopy or pHRodo for Incucyte live imaging, and 5 POSs/cell were added to the cells. For Opera microscopy, the cells were washed three times with PBS and fixed with 4% PFA, then counterstained with Hoechst and phalloidin. For Incucyte live evaluation, images were taken every 2 h for 24 h. Then, cells were washed to monitor POS degradation followed by imaging every 2 h for additional 48 h.

### Seahorse assay

Agilent Seahorse XF Cell Mito Stress Test Kit (103015-100) was used according to manufacturer’s instructions. 80,000 iPSC-RPE cells were plated in GFR Matrigel-coated Seahorse XFe96 FluxPak (102416-100) plates and cultured for 2 weeks. 25 mM glucose, 1 mM pyruvate, and 2 mM glutamine were added to assay medium. During the assay, 2.5 μM Oligomycin A, 2 μM FCCP, and 0.5 μM Rotenone/Antimycin A (Rot/AA) were used. An average of the three consecutive measurements was taken to calculate baseline OCR, lowest OCR after oligomycin, maximal OCR after FCCP, and minimum OCR after Rot/AA. The non-mitochondrial OCR (pmol/min) value is the minimum OCR after Rot/AA. Basal respiration rate (pmol/min) is the baseline OCR minus minimum OCR after Rot/AA. Maximal respiration rate (pmol/min) is the maximal OCR after FCCP minus the minimum OCR after Rot/AA. Proton leak (pmol/min) is the lowest OCR after oligomycin minus the minimum OCR after Rot/AA. ATP production is equivalent to baseline OCR minus the lowest OCR after oligomycin. The spare respiratory capacity is equivalent to maximal respiration rate minus the baseline OCR. The coupling efficiency is equivalent to 100^∗^(ATP production/baseline OCR). MitoATP production rate (pmol ATP/min) is equivalent to ATP production^∗^2^∗^2.75.

### A2E and blue light challenge model

A2E was synthesized by Boehringer Ingelheim according to previously described protocols (Parish et al., 1998). A2E concentrations were tested to determine the dose that induces inflammation but not cell death, using TEER measurement, TUNEL assay, and MSD proinflammatory ELISA panel. Cells were exposed to selected A2E concentration (10 μM) for 3 days followed by blue light exposure for 10 min on the fourth day. Five treatments were performed on each line: untreated, DMSO, DMSO with blue light, A2E, and A2E with blue light. On the fifth day, supernatant and cells were collected for ELISA and RNA-seq, respectively. Cytokine ELISA and TEER measurement methods can be found in supplementary methods.

### Whole-transcriptomics analysis

RNA isolation and quality control and mRNA sequencing methods can be found in supplementary methods. Sequencing reads from the RNA-seq experiment were processed with a pipeline built upon the ENCODE’ “Long RNA-seq” pipeline: filtered reads were mapped against the *Homo sapiens* (human) genome hg38/GRCh38 (primary assembly, excluding alternate contigs) using the STAR aligner (STAR version 2.5.2b) allowing for soft clipping of adapter sequences. For quantification of transcript levels, the Ensembl version 86 annotation files were used, corresponding to GENCODE 25. Annotated samples were quantified using RSEM (RSEM version 1.3.0) and featureCount (featureCount version 1.5.1). Quality controls were implemented using FastQC (FastQC version 0.11.5), picardmetrics (picardmetrics version 0.2.4), and dupRadar (dupRadar version 1.0.0) at the respective steps.

Normalized log2 counts per million mRNA expression values were calculated using the voom function provided by the limma R package (limma version 3.44.3). Initial quality control was performed including principal component analysis. This revealed that samples from the first differentiation round of donor 53 were outliers compared to the remaining samples. Additional confounding test between principal components and sample quality as well as sequencing parameters did not suggest any further need for exclusions or corrections. All samples had a very high RIN (>9.9). We proceeded with downstream analysis using the limma package for differential expression. The pathview package was used for plotting KEGG pathway maps (Luo and Brouwer, 2013). All plots were generated using ggplot. Analyses were performed in R version 4.0.2.

### Cholesterol efflux

Cholesterol efflux experiments were performed on RPE cells cultured on white 96 plates (PerkinElmer, 6005181), which are suitable for radioactive cholesterol measurement, for 14 days. RPE cells were washed with PBS with 0.2% BSA (Serva, 11945.03), and 100 μL of RPE media containing 0.2% BSA and radioactive-labeled cholesterol[4-14C] (Biotrend, ART0255) at a concentration of 0.2 μCi/100 μL were added. Cells were then incubated for 24 h at 37°C in 5% CO_2_. Next, cells were washed twice with PBS and 0.2% BSA and incubated with RPE media containing HDL (500 μg/mL; Abeomics, 32–3944) for 6 h. After 6 h, 30 μL of supernatant was transferred into white bottom plates containing 240 μL MicroScint 20 (PerkinElmer, 6013621). Plates were sealed and incubated for 60 min at room temperature while shaking. Simultaneously, the cells were washed twice with PBS and 0.2% BSA and lysed with 30 μL 0.1 M NaOH containing 0.1% SDS for 30 min. Then, 240 μL MicroScint 20 was added on top, and plates were incubated for 60 min in the dark at room temperature while shaking. Cholesterol in supernatant and lysate was quantified using Topcount. The percentage of effluxed cholesterol was calculated:

$$\%\;Cholesterol\;efflux=\frac{CPM\;in\;Supernatant\;}{CPM\;in\;Supernatant+Lysate}x\;100$$

The AAV6.2 capsid was chosen based on its high transduction efficiency *in vitro*, including human RPE cells (Weinmann et al., 2022). Vectors expressing miR-E-based shRNAs targeting *CFH* were produced and purified as described previously (Strobel et al., 2019). AAVs were transduced to primary human RPE cells (Clonetics Human RPE Cell Systems), at a multiplicity of infection (MOI) of 10, 50, 100, and 200, 7 days post seeding and incubated for 7 additional days before knockdown efficiency was measured via qPCR. For the cholesterol efflux experiment, 50 MOI was chosen, and the experiment was similarly performed 7 days post transduction.

### Human serum challenge and MAC deposition

RPE cells, grown on 96 well plates, were treated with 10% human serum for 24 h, then washed with PBS and fixed with 4% PFA. Cells were stained with C5b-9 (Biomol, C7850-26.100) and counterstained with phalloidin and Hoechst and imaged with Opera confocal microscope.

### Western blot FH

FH western blot was performed using SallySue (12–230 kDa) size separation master kit (Bio-techne, SM-S001) and SallySue detection kit (Bio-techne, DM-001) with anti-FH (Thermo Fisher Scientific, MA5-32714), according to manufacturer’s instructions. Cells were lysed in RIPA buffer, and protein concentration was determined with BCA kit (Thermo Fisher Scientific, 23225) before loading 0.2 mg/mL into the capillaries.

### Transmission electron microscopy

Cells on transwells were fixed in 2% glutaraldehyde in 0.1 M Na-cacodylate buffer for 72 h, then rinsed with 0.1 M Na-cacodylate buffer for 1 h. Next, they were fixed with 2% OsO4 solution for 1 h and rinsed again with 0.1 M Na-cacodylate buffer. Sample preparation was performed using AC20 (Leica) as follows: 3 x ethanol 70%, 3 x ethanol 90%, 3 x ethanol absolute, 3 x propylene oxide, 1 x mixture Epon/propyleneoxide1 = 1:3, 1 x mixture Epon/propyleneoxide2 = 1:1, 1 x pure Epon. Samples were embedded in molds with pure Epon and placed in the incubator with increasing temperatures from 45°C to 67°C for polymerization. Epon blocks are cut using the UC7, ultramicrotome (Leica). First semi-thin coupes of 1 μm are cut and colored using a toluidine blue 1% solutions to check for cells. Afterward, 80 nm ultra-thin coupes are cut and placed on grids for transmission electron microscopy imaging (JEM1400 JEOL electron microscope). Around 12 to 16 fields with 1–4 cells were taken from each donor at 1 month and 3 months time points. Cells and apical/basal deposits were counted per field, and a ratio of deposits to cell number was plotted.

### Statistical analysis

All key experiments were performed with three consecutive differentiation rounds and three technical repeats per condition and per line. For significance calculation, the ANOVA function in GraphPad Prism was used for all experiments except for whole-transcriptomics analysis.

## Resource availability

### Lead contact

For further information inquire with Seba Almedawar (seba.almedawar@boehringer-ingelheim.com)

### Materials availability

iPSC lines used in this paper are deposited in Database: hPSCreg (https://hpscreg.eu/) as lines: SCTCi010-A (RRID:CVCL_A2YE), SCTCi008-A (RRID:CVCL_A2YI), SCTCi009-A (RRID:CVCL_A2YD), SCTCi011-A (RRID:CVCL_A2YF), SCTCi012-A (RRID:CVCL_A2YG), and SCTCi013-A (RRID:CVCL_A2YH).

### Data and code availability

The accession number for the RNA-seq data reported in this paper are available in the GEO Database: GSE275494.

## Acknowledgments

This work was funded by 10.13039/501100003246NWO (VICI 91817601), Health Holland (LSH-TKI LSHM19001), the Dutch Society for the Replacement of Animal Testing and Boehringer Ingelheim Pharma GmbH & Co. KG. We would like to thank the scientists from the Pathology department at RUMC especially Luuk Reuvers and the scientists from the GCBDS sequencing facility at Boehringer Ingelheim especially Dr. Elke Markert. We thank Dr. Benjamin Strobel at Boehringer Ingelheim for generating the AAVs. We also thank scientists at RUMC and Boehringer Ingelheim who contributed to the establishment of assays used in this work including Andrea Lorenz, Eva Bernstein, Dr. Giuliana Gagliardi, and Anita de Breuk.

## Author contributions

Conceptualization and project supervision, R.A.B., S.A., A.I.d.H., and C.C.W.K.; manuscript writing, S.A. and S.C.A.B.; patient iPSC derivation and RPE differentiation, L.K.; experimental work, S.A., L.K., and S.C.A.B.; funding acquisition, R.A.B. and A.I.d.H.; manuscript review and editing, all authors.

## Declaration of interests

S.A. and R.A.B. are employees of Boehringer Ingelheim. A.I.d.H. is an employee of AbbVie. The study was partially funded by Boehringer Ingelheim.

## Supplemental information

Document S1. Figures S1–S6, Table S1, and Supplementary experimental procedures

Document S2. Article plus supplemental information
