# Supplementary material for: Non-canonical roles of CFH in retinal pigment epithelial cells revealed by dysfunctional rare CFH variants
Source: Stem Cell Reports. 2025 Jan 2;20(1):102385. doi: 10.1016/j.stemcr.2024.11.015 (PMC11784488; doi:10.1016/j.stemcr.2024.11.015)
Supplement: Document S1. Figures S1–S6, Table S1, and Supplementary experimental procedures [file mmc1.pdf]

Stem Cell Reports, Volume *20*

Supplemental Information

Non-canonical roles of *CFH* in retinal pigment epithelial cells revealed by dysfunctional rare *CFH* variants

Sofie C.A. Brink, Louet Koolen, Caroline C.W. Klaver, Remko A. Bakker, Anneke I. den Hollander, and Seba Almedawar

Supplementary Figures and Legends

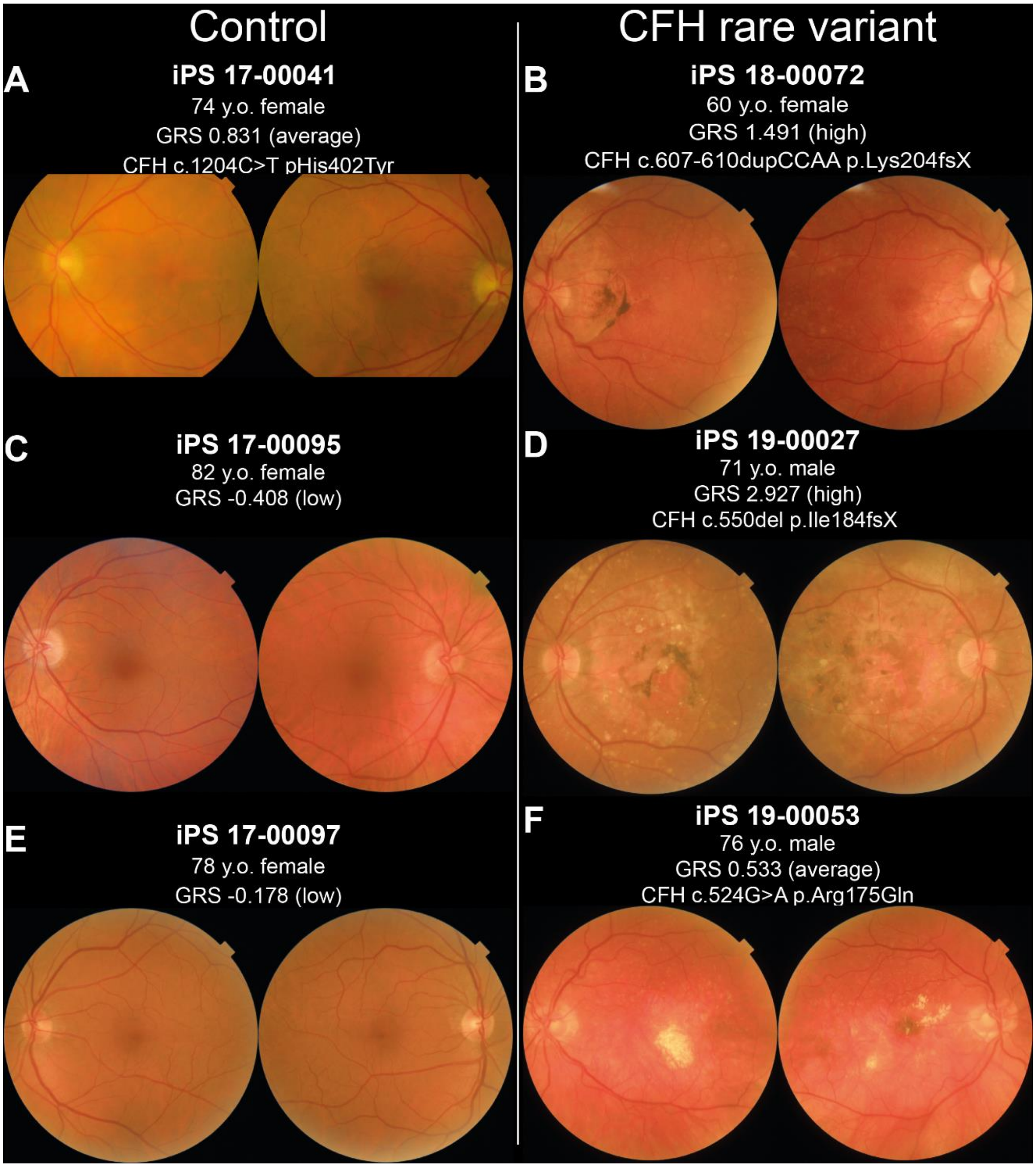

Supplementary Figure 1. Summary of donor information, including for each line the donor age, gender, GRS based on the Fritsche et al. (2016) SNPs, mutation and color fundus photograph (CFP). Lines 41, 97 and 95 are healthy controls (A, C, E). Line 72 was diagnosed with a peripapillary MNV membrane in the left eye, and serous detachment of the RPE and intraretinal fluid due to MNV in the right eye (B). Line 27 was diagnosed with bilateral end-stage AMD with GA in the macula and a fibrous scar due to MNV (D). Line 53 was diagnosed with GA and an old MNV membrane in the right eye and GA in the left eye (F). Detailed Clinical information in Table S1.

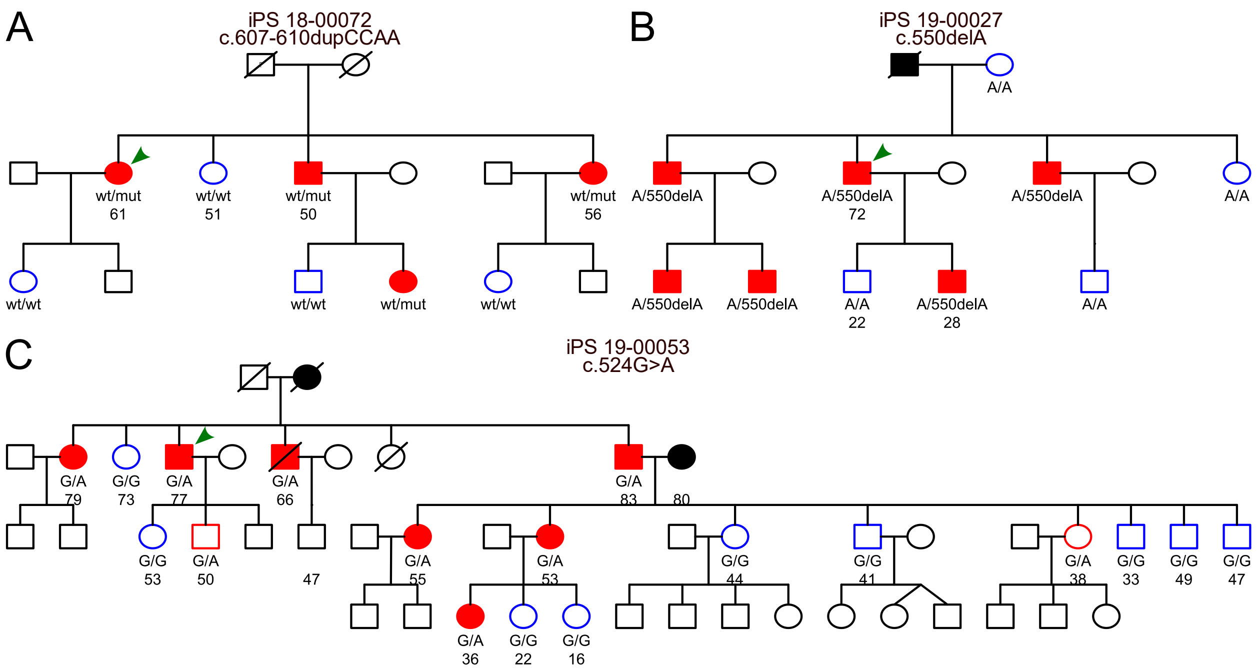

Supplementary Figure 2. Pedigrees displaying known genotypes and ages below each individual. Indicating men (square) and women (circle). Phenotypes are indicated by filling of shapes AMD (filled) or no AMD (empty). Genotypes are indicated in text and color: variant carriers (red), no variant (blue), unknown genotype (black). Green arrow denotes cell line donor. Families of donors of lines 72 and 27 segregate (A & B), whereas the family of line 53 displays incomplete penetrance (C).

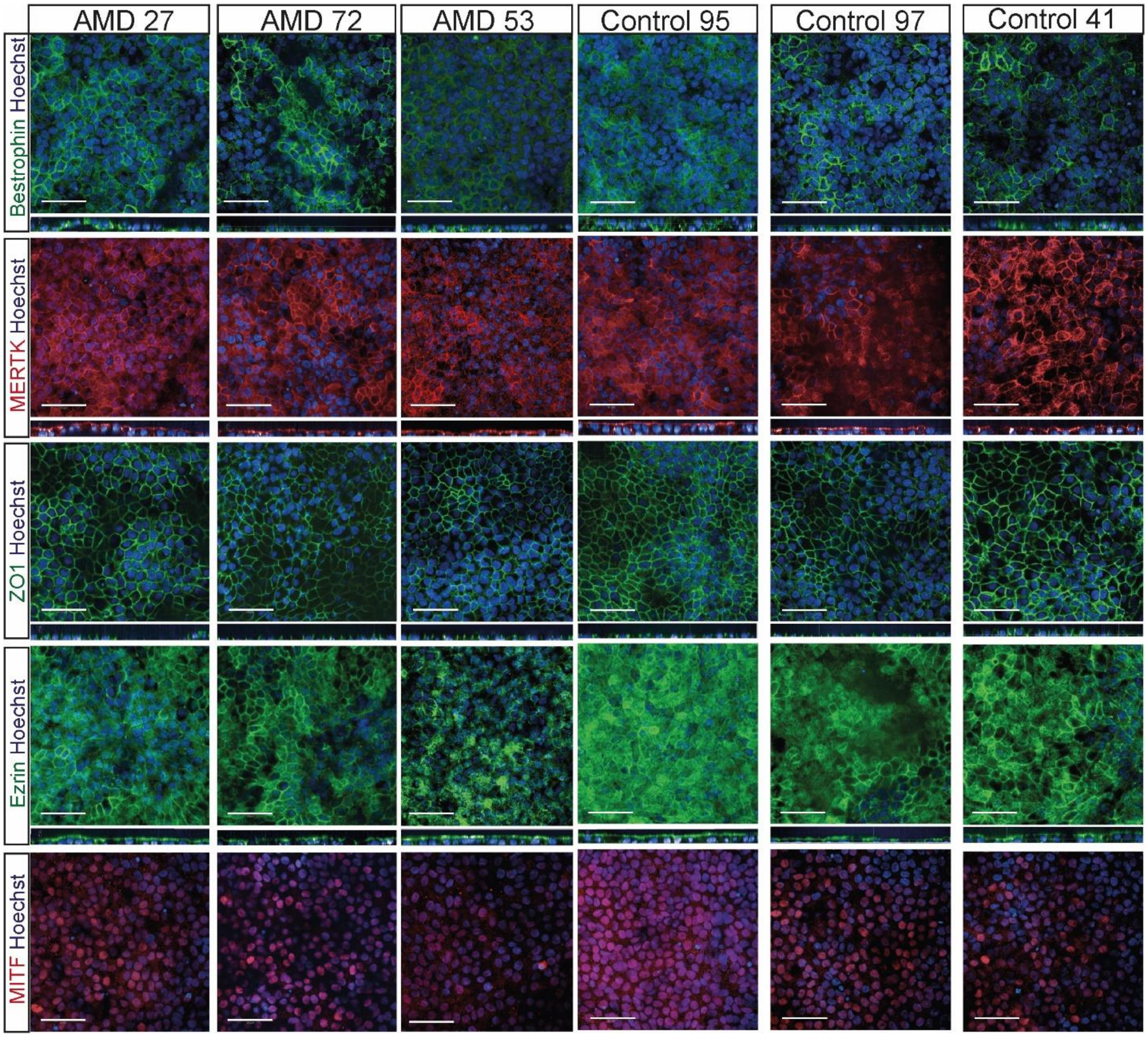

Supplementary Figure 3. Immunofluorescence analysis of RPE markers in iPSC-RPE cells. Scale bar: 50 µm.

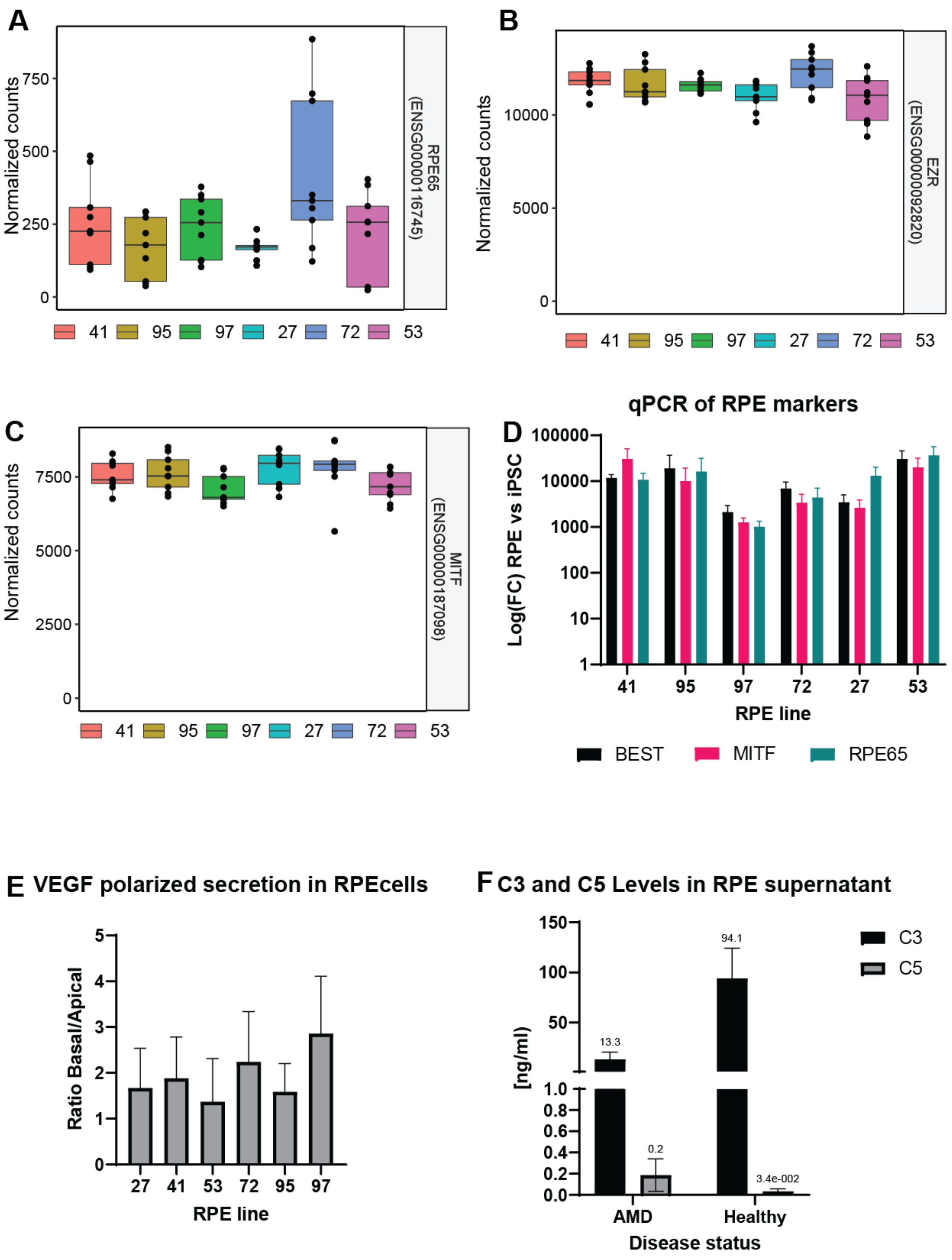

Supplementary Figure 4. A-C. Expression of RPE specific markers on the mRNA levels (derived from the NGS data set). D. Expression of RPE specific markers on the mRNA levels (RT-qPCR). E. VEGF polarized secretion of RPE cultured in transwells. No

significant difference in the measurements was observed between the lines in A-E. F. C3 and C5 secreted levels of RPE cells cultured in transwells. N=3 differentiation rounds. Error bars represent mean ± SD.

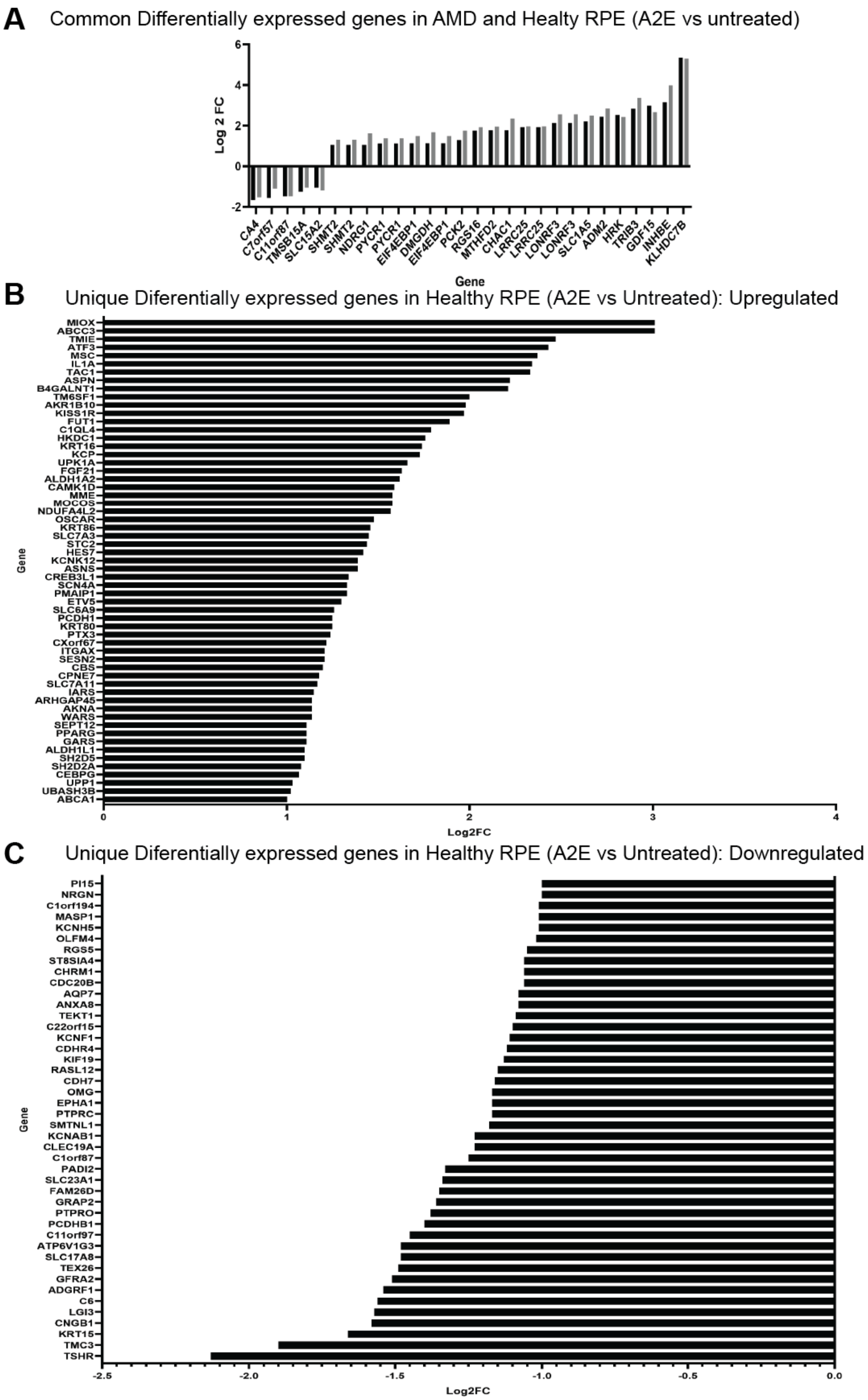
A
Common Differentially expressed genes in AMD and Healty RPE (A2E vs untreated)
Log 2 FC
6
4
2
0
-2
CA4
C7orf57
C11orf87
TMSB15A
SLC15A2
SHMT2
SHMT2
NDRG1
PYCR1
PYCR1
EIF4EBP1
DMGDH
EIF4EBP1
PCK2
RGS16
MTHFD2
CHAC1
LRRC25
LRRC25
LONRF3
LONRF3
SLC1A5
ADM2
HRK
TRIB3
GDF15
INHBE
KLHDC7B
Gene
B
Unique Diferentially expressed genes in Healthy RPE (A2E vs Untreated): Upregulated
Gene
MIOX
ABCC3
TMIE
ATF3
MSC
IL1A
TAC1
ASPN
B4GALNT1
TM6SF1
AKR1B10
KISS1R
FUT1
C1QL4
HKDC1
KRT16
KCP
UPK1A
FGF21
ALDH1A2
CAMK1D
MME
MOCOS
NDUFA4L2
OSCAR
KRT86
SLC7A3
STC2
HES7
KCNK12
ASNS
CREB3L1
SCN4A
PMAIP1
ETV5
SLC6A9
PCDH1
KRT80
PTX3
CXorf67
ITGAX
SESN2
CBS
CPNE7
SLC7A11
IARS
ARHGAP45
AKNA
WARS
SEPT12
PPARG
GARS
ALDH1L1
SH2D5
SH2D2A
CEBPG
UPP1
UBASH3B
ABCA1
0
1
2
3
4
Log2FC
C
Unique Diferentially expressed genes in Healthy RPE (A2E vs Untreated): Downregulated
Gene
PI15
NRGN
C1orf194
MASP1
KCNH5
OLFM4
RGS5
ST8SIA4
CHRM1
CDC20B
AQP7
ANXA8
TEKT1
C22orf15
KCNF1
CDHR4
KIF19
RASL12
CDH7
OMG
EPHA1
PTPRC
SMTNL1
KCNAB1
CLEC19A
C1orf87
PADI2
SLC23A1
FAM26D
GRAP2
PTPRO
PCDHB1
C11orf97
ATP6V1G3
SLC17A8
TEX26
GFRA2
ADGRF1
C6
LGI3
CNGB1
KRT15
TMC3
TSHR
-2.5
-2.0
-1.5
-1.0
-0.5
0.0
Log2FC

Supplementary Figure 5. Expression of significant (P-value<0.01) differentially expressed genes (mRNA derived from NGS data set). A. Common differentially expressed genes in AMD and Healthy RPE (A2E vs untreated). B. Unique differentially expressed genes in Healthy RPE (A2E vs untreated) Upregulated. C. Unique differentially expressed genes in Healthy RPE (A2E vs untreated) downregulated. N=3 differentiation rounds.

A2E+ Blue Light vs Untreated: AMD RPE

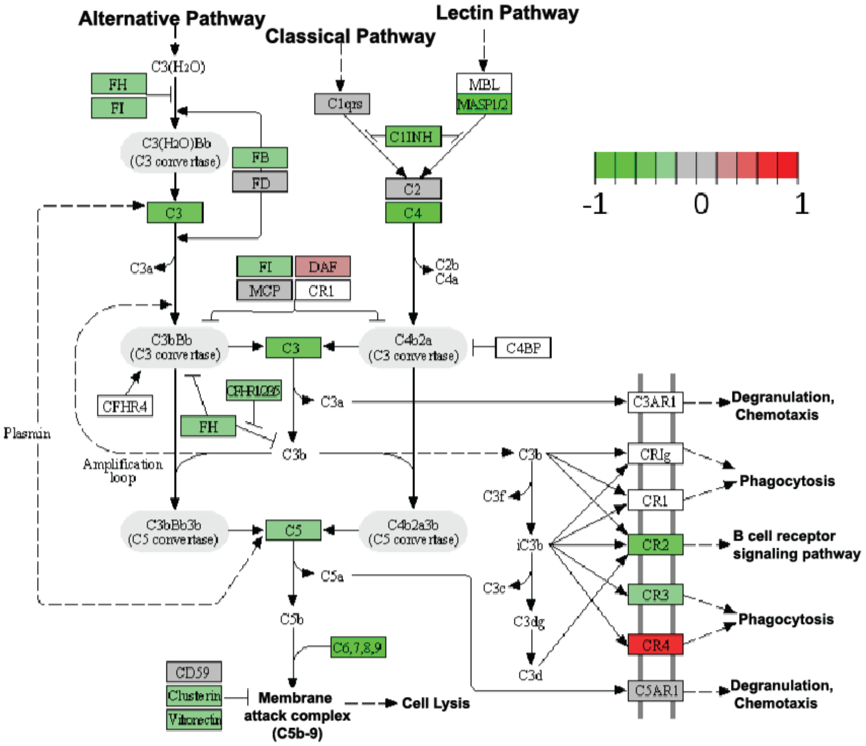

A2E+ Blue Light vs Untreated: Control RPE

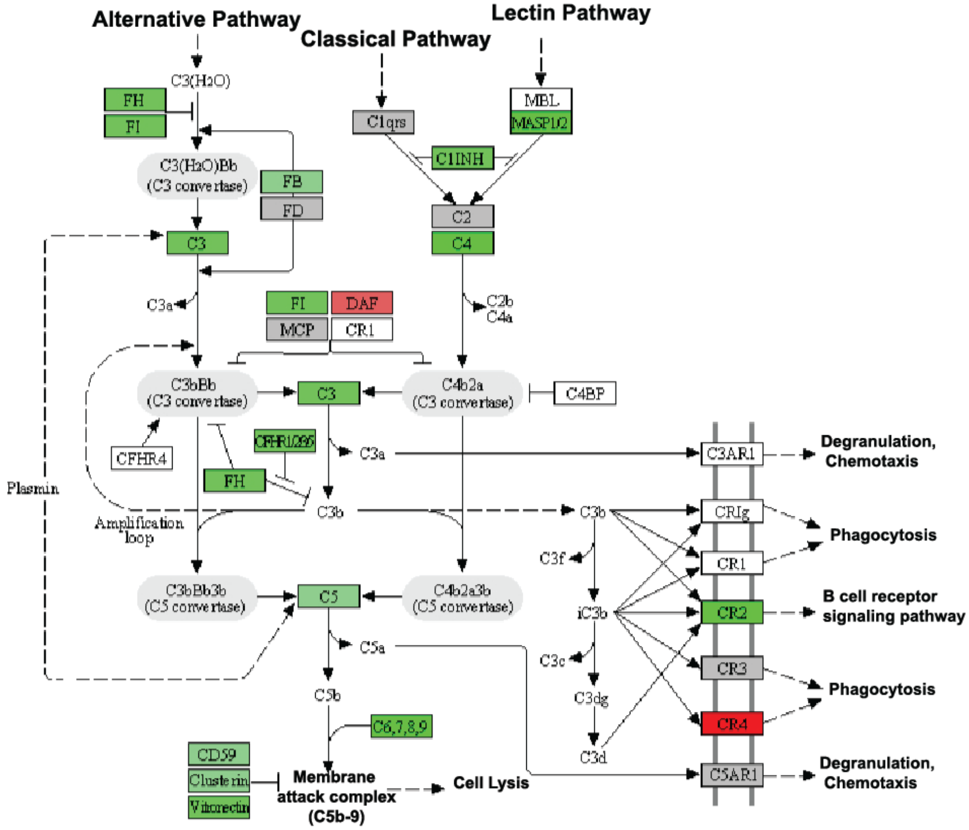

Supplementary Figure 6. RNA-seq changes in expression of the complement pathway in CFH variant lines and controls under A2E challenge vs untreated (mRNA derived from NGS data set). N= 3 differentiation rounds. Only DEG with P-Value<0.01 are represented in green or red.

Supplementary Tables

Table 1 Potential disease-causing SNPs in the CFH gene. iPSC lines description

**iPSC line**	**Mutation**	**Phenotype**	**GRS***
IPS18-00072 (SCTCi011-A)	*CFH* c.607-610dupCCAA p.Lys204fsX*24 (heterozygous) Frameshift CCP 3 with stop in CCP 4	60-year old female with advanced AMD in both eyes. A peripapillary choroidal neovascular membrane is present in the left eye, and color fundus photographs (CFP) and optical coherence tomography (OCT) images of the right eye show drusen, serous detachment of the retinal pigment epithelium and some intraretinal fluid due to a choroidal neovascularization.	1.491 (high)
IPS19-00027 (SCTCi012-A)	*CFH* c.550del p.Ile184fsX*32 (heterozygous) Frameshift CCP 3 with stop in CCP 4	71-year-old male with end stage AMD in both eyes. Color fundus photographs (CFP) and optical coherence tomography (OCT) images show atrophy in the macula and a fibrous scar due to choroidal neovascularization.	2.927 (high)
IPS19-00053 (SCTCi013-A)	*CFH* c.524G>A p.Arg175Gln (heterozygous) Missense CCP 3 (C3b binding domain)	76-year-old male with advanced AMD in both eyes. In the right eye geographic atrophy and an old choroidal neovascular membrane is present. Color fundus photographs (CFP) and optical coherence tomography (OCT)	0.533 (average)

		images of the left eye show drusen and geographic atrophy.	
IPS17-00041 (SCTCi010-A)	*CFH* c.1204C>T ; p.His402Tyr (heterozygous) Missense CCP 7	-	0.831 (average)
IPS17-00095 (SCTCi008-A)	-	-	-0.408 (low)
IPS17-00097 (SCTCi009-A)	-	-	-0.178 (low)

GRS: Genetic risk score based on effect sizes from Fritsche *et al.* (2016).
CCP: Complement control protein domain of FH.

Supplementary Experimental Procedures

Human Vascular Endothelial Growth Factor (VEGF) DuoSet ELISA

Human VEGF DuoSet ELISA kit (R&D Systems, DY1177-05 and DY008) was used according to the manufacturer's instructions. At 1 month of culture, supernatants were collected from both the apical and the basolateral chambers of the transwells and stored at –20 °C. The supernatants were diluted 1:1 and the procedure was repeated across three RPE differentiation rounds.

Real-Time Quantitative PCR

RNA isolation and reverse transcription was performed using NucleoSpin RNA isolation kit (Macherey-Nagel, 740955.250) and iScript cDNA synthesis kit (Bio Rad, 1708891) according to manufacturers instructions. Real-Time quantitative PCR was performed using TaqMan Fast Advanced Master Mix (Life Technologies; 4444965) according to manufacturer instructions, using the following primersets: 18s primers Hs99999901_s1 (Thermo Fisher Scientific; 4453320), BEST1 primers Hs00188249_m1 (Thermo Fisher Scientific; 4453320), CFH primers Hs00962373_m1 (Thermo Fisher Scientific; 4331182), MERTK primers Hs01031973_m1 (Thermo Fisher Scientific; 4453320), MITF primers Hs01117294_m1 (Thermo Fisher Scientific; 4453320), PEDF primers Hs01106934_m1 (Thermo Fisher Scientific; 4448892, RPE65 primers Hs01071462_m1 (Thermo Fisher Scientific; 4453320), and VEGF primers Hs00900055_m1 (Thermo Fisher Scientific; 4453320). mRNA was isolated from three RPE differentiation rounds and two batches of cDNA were reverse transcribed from isolated mRNA and 4 technical repeats were performed from each sample. CT values from the different genes were normalized to housekeeping genes CT values (18S or GAPDH).

Immuno-fluorescence and Microscopy

After blocking and permeabilization for one hour at room temperature using PBS buffer containing 0.5% BSA and 0.3% TritonX, primary antibodies were left overnight at 4°C in blocking buffer, diluted as follows: MITF (1:200; Abcam ab122982), MERTK (1:1000; Abcam ab52968), bestrophin (1:500; Abcam ab2182), ZO-1 (1:200; Invitrogen 402200), and EZRIN (1:200; Diagnostic BioSystems Mob380). Cells were washed three times with PBS and incubated with the secondary antibody for two hours at room temperature. Finally, they were imaged with the Opera high-throughput confocal microscope.

Photoreceptor outer segments (POS) Isolation

POS were isolated from porcine eyes as described in Almedawar *et al.* (2020). Briefly, retinal tissue was isolated from 50 porcine eyes, isolated retinas were shaken thoroughly in homogenization buffer (20% sucrose, 20mM Tris Acetate, 2mM MgCl2, 10 mM glucose, and 5 mM Taurine), then filtered through double layers of gauze as originally described (Molday *et al.* 1987). The filtrate was split in 6 tubes each containing a sucrose gradient of 27%, 33%, 41%, 50% and 60%, and centrifuged at 28 000 rpm for one hour at 4°C. The faint orange band was collected from each gradient, and diluted 5x with wash buffer (10% sucrose, 20mM sodium phosphate buffer and 5 mM Taurine), and centrifuged at 4800 rpm for ten minutes. Finally, the pellets were resuspended with POS storage solution (DMEM with 2.5% sucrose) and stored at -80°C.

POS labeling

Alexa Fluor 488 (AF488) or pHRodo were added to the POS after thawing for one hour at 25°C with shaking (500 rpm). POS were next centrifuged at 3600 rcf for 5 minutes and pellets were resuspended with the washing buffer twice (10% sucrose, 20 mM phosphate buffer pH7.2 and 5 mM taurine). POS were counted with LUNA cell counter.

ELISA MSD-Proinflammatory Cytokine Panel

ELISA was performed in triplicate across three RPE differentiation rounds using V-PLEX Proinflammatory Panel1 (human) Kit (Meso Scale Discovery, K15049D-2) according to manufacturer's instructions. Supernatants of cells treated with A2E with blue light or DMSO (vehicle) were collected and stored at -80 °C until measured.

TEER Measurement

TEER was measured with the EVOM2 using STX2 electrodes across three RPE differentiation rounds before A2E or DMSO treatment, or before cell lysis at day 5. Reported TEER values were calculated as follows:

$$TEER\ (\Omega * \mathrm{cm}^2) = \left(Sample\ TEER\ (\Omega) - Blank\ TEER\ (\Omega)\right) x\ Area\ cm^2$$

Alternatively, CellZScope was used for continuous measurement of TEER before and following A2E treatment.

RNA isolation and Quality Control

RPE cells, were lysed in RLT buffer containing 1% ß-Mercaptoethanol. Total RNA was extracted using phenol chloroform-based extraction and subsequently isolated with the MagMAX-96 Total RNA Isolation Kit (Thermo Fisher Scientific, AM1830), with DNase digestion prior to final elution (Qiagen, 79254). Total RNA samples were assessed using both the fluorescence-based Broad Range Quant-iT RNA Assay Kit (Thermo Fisher Scientific) and the Standard Sensitivity RNA Analysis DNF-471 Kit on a 96-channel Fragment Analyzer (Agilent). Concentrations averaged at 137.9 ng/µL while RIN ranged from 9.0 to 10.0, with a median at 10.0. 9 replicates were run for each condition.

mRNA sequencing

90 human RPE-derived RNA samples were normalized on the MicroLab STAR automated liquid platform (Hamilton). The library was constructed with 250ng of total RNA using the NEBNext Ultra II Directional RNA Library Prep Kit for Illumina #E7760, the NEBNext Poly(A) mRNA Magnetic Isolation Module #E7490 upstream and the NEBNext Multiplex Oligos for Illumina #E7600 downstream (all New England Biolabs). The only deviation from the manufacturer's protocol was the use of Ampure XP beads (Beckman Coulter) for double-stranded cDNA purification, instead of SPRIselect Beads. The index PCR was performed with 12 cycles. The final library was eluted in 25µL. mRNA libraries were then quantified by the High Sensitivity dsDNA Quanti-iT Assay Kit (ThermoFisher) on a Synergy HTX (BioTek). Library molarity averaged at 154 nM. Smear analysis of mRNA libraries verified size distribution (361bp average) and adapter dimer presence (<0.5%) was verified by the High Sensitivity Small Fragment DNF-477 Kit on a 96-channel Fragment Analyzer (Agilent). All 90 sequencing libraries were normalized on the MicroLab STAR (Hamilton), pooled and spiked in with PhiX Control v3 (Illumina). The library pool was clustered on an S4 Flow Cell and sequenced on a NovaSeq 6000 Sequencing System (Illumina) with dual index, paired-end reads at 2 x 100 bp length (Read parameters: Rd1: 101, Rd2: 8, Rd3: 8, Rd4: 101), reaching an average depth of 28.4 million Pass-Filter reads per sample (7.9% CV).

References

Almedawar, Seba, Katerina Vafia, Sven Schreiter, Katrin Neumann, Shahryar Khattak, Thomas Kurth, Marius Ader, Mike O. Karl, Stephen H. Tsang, and Elly M. Tanaka. 2020. 'MERTK-Dependent Ensheathment of Photoreceptor Outer Segments by Human Pluripotent Stem Cell-Derived Retinal Pigment Epithelium', *Stem Cell Reports*, 14: 374-89.

Fritsche, Lars G., Wilmar Igl, Jessica N. Cooke Bailey, Felix Grassmann, Sebanti Sengupta, Jennifer L. Bragg-Gresham, Kathryn P. Burdon, et al. 2016. 'A large genome-wide association study of age-related macular degeneration highlights contributions of rare and common variants', *Nature Genetics*, 48: 134-43.

Molday, R S, D Hicks, and L Molday. 1987. 'Peripherin. A rim-specific membrane protein of rod outer segment discs', *Investigative Ophthalmology & Visual Science*, 28: 50-61.
